# Unraveling the Link between Periodontitis and Coronavirus Disease 2019: Exploring Pathogenic Pathways and Clinical Implications

**DOI:** 10.3390/biomedicines11102789

**Published:** 2023-10-14

**Authors:** En-Chin Lin, Yi-Chun Chiang, Hsuan-Yu Lin, Shao-Yu Tseng, Yu-Ting Hsieh, Jer-An Shieh, Yu-Hao Huang, Hsiang-Tai Tsai, Sheng-Wei Feng, Tzu-Yu Peng, I-Ta Lee

**Affiliations:** School of Dentistry, College of Oral Medicine, Taipei Medical University, Taipei 110, Taiwan; b202111093@tmu.edu.tw (E.-C.L.); b202111011@tmu.edu.tw (Y.-C.C.); b202111063@tmu.edu.tw (H.-Y.L.); b202111061@tmu.edu.tw (S.-Y.T.); b202111056@tmu.edu.tw (Y.-T.H.); b202111017@tmu.edu.tw (J.-A.S.); b202111079@tmu.edu.tw (Y.-H.H.); b202111081@tmu.edu.tw (H.-T.T.); shengwei@tmu.edu.tw (S.-W.F.)

**Keywords:** periodontitis, coronavirus disease 2019, severe acute respiratory syndrome coronavirus 2, angiotensin-converting enzyme 2, transmembrane serine protease 2

## Abstract

Periodontitis involves the inflammation of the periodontal tissue, leading to tissue loss, while coronavirus disease 2019 (COVID-19) is a highly transmissible respiratory disease caused by the severe acute respiratory syndrome coronavirus 2 (SARS-CoV-2), which is amplified by poor systemic health. Key facilitators of SARS-CoV-2’s entry into host cells are angiotensin-converting enzyme 2 (ACE2) and transmembrane serine protease 2 (TMPRSS2). This review reveals that periodontal pockets can serve as a hotspot for virus accumulation, rendering surrounding epithelia more susceptible to infection. Given that ACE2 is expressed in oral mucosa, it is reasonable to suggest that poor periodontal health could increase the risk of COVID-19 infection. However, recent studies have not provided sufficient evidence to imply a significant effect of COVID-19 on periodontal health, necessitating further and more long-term investigations. Nevertheless, there are hypotheses linking the mechanisms of the two diseases, such as the involvement of interleukin-17 (IL-17). Elevated IL-17 levels are observed in both COVID-19 and periodontitis, leading to increased osteoclast activity and bone resorption. Lastly, bidirectional relationships between periodontitis and systemic diseases like diabetes are acknowledged. Given that COVID-19 symptoms may worsen with these conditions, maintaining good oral health and managing systemic diseases are suggested as potential ways to protect against COVID-19.

## 1. Introduction

In the intricate realm of human health, periodontitis stands as a formidable adversary, an inflammatory juggernaut wreaking havoc within the oral cavity. Its impact extends beyond mere discomfort; it corrodes the very foundations of dental health, leading to the destruction of crucial structures like the periodontal ligament and alveolar bone [1]. The alarmingly high prevalence, with approximately 11.2% of adults worldwide afflicted between 1990 and 2010, paints a stark reality—one in ten individuals grapple with this condition, emphasizing the urgency for understanding and prevention [2]. The risk factors, such as smoking and obesity, which are often self-inflicted, add a layer of preventability to this oral menace, emphasizing the importance of lifestyle choices in maintaining oral health [3]. The classification system introduced in 1999 further delineates periodontitis into distinct forms, offering clinicians a nuanced understanding ranging from necrotizing and chronic periodontitis to aggressive manifestations linked with systemic diseases [4]. Amid this landscape of oral challenges, a new global health threat emerged: coronavirus disease 2019 (COVID-19), caused by the relentless severe acute respiratory syndrome coronavirus 2 (SARS-CoV-2). Its rapid spread, facilitated by human-to-human transmission via droplets, transformed it into a pandemic, impacting millions across the globe [5]. By June 2020, the world had witnessed over 7 million infections and a staggering toll of more than 400,000 lives lost, underscoring the urgency of understanding this novel viral adversary [6]. Its diverse array of symptoms, from the loss of taste and smell (ageusia and anosmia) to severe respiratory distress, added layers of complexity to the clinical presentation, challenging healthcare systems worldwide [7]. In the crucible of research, where scientific curiosity meets the pressing needs of public health, a compelling question emerges: how do these seemingly disparate entities, periodontitis, and COVID-19, intertwine within the human body? Researchers, akin to explorers navigating uncharted territories, are delving into this intricate interplay. What emerges is a revelation: periodontitis is not an isolated oral woe but a condition with profound systemic implications, weaving connections with diseases like cardiovascular ailments [8]. The focus of contemporary research extends beyond mere coexistence. Scientists are peering into the depths of cellular interactions and immunological responses, unraveling the intricate mechanisms wherein both COVID-19 and periodontitis might fuel each other’s impact. Does the inflammation orchestrated by periodontitis serve as a welcome mat for the SARS-CoV-2, amplifying its virulence within the body? Or perhaps, does the viral invasion, in turn, exacerbate the oral inflammation, leading to a vicious cycle of health complications? Understanding these multifaceted interactions is not merely an academic pursuit—it is a crucial endeavor that promises significant strides in patient management. Insights gained from deciphering the dialogue between COVID-19 and periodontitis could revolutionize treatments and preventive strategies, offering a beacon of hope for the countless individuals affected by this intricate interplay of diseases. In the ever-evolving saga of global health, the narrative of periodontitis and COVID-19 represents a pivotal chapter, one where scientific inquiry meets the pressing demands of public health. As research strides forward, each discovery serves as a milestone, illuminating the path toward a future where these intertwined challenges are met with knowledge, understanding, and ultimately, effective solutions.

## 2. Molecular Pathogenesis of Periodontitis: From Plaque Accumulation to Gene Expression Insights

Periodontitis, a prevalent oral disease, exerts its detrimental effects on individuals predisposed to specific risk factors, leading to irreversible damage to tooth-supporting tissues [1,9]. At its core, the disease originates from the insidious accumulation of dental plaque or biofilm on tooth surfaces, serving as the epicenter of molecular warfare between health and disease [1]. Within the microenvironment of periodontal tissues, striking disparities exist between individuals untouched by infection and those ensnared by the clutches of periodontitis [10]. The discerning sentinel in this context is the gingival crevicular fluid (GCF). The molecular composition of GCF reflects the internal landscape of periodontal health, encompassing proteins, proteinases, cytokines, and chemokines, presenting diverse and dynamic molecular signatures in the presence of periodontal disease [11]. Within the diseased pockets of periodontitis, particularly intriguing is the presence of various viral species, including the notorious SARS-CoV-2, finding sanctuary [12]. The genesis of periodontitis lies in the relentless replication of bacteria on tooth surfaces, forming the seemingly innocuous dental plaque. Chemical substances released by these bacteria orchestrate a symphony of destruction within the periodontium, simultaneously activating the immune system, triggering inflammatory responses, and leading to the breakdown of surrounding tissues [9]. In this intricate web of immune and molecular interactions, chemokines play pivotal roles, orchestrating the recruitment and activation of inflammatory cells, including macrophages, neutrophils, and lymphocytes, exacerbating the disease progression [13]. Simultaneously, a subtle player, cathepsin S (CTSS), a cysteine protease, lurks in the shadows. CTSS potentially inhibits autophagy, disrupting cellular survival mechanisms, further intensifying the progression of periodontitis [14]. In the realm of severe cases, periodontitis leaves an indelible mark—irreversible loss of alveolar bone, often extending its impact to systemic osteoporosis in distant parts of the body [9,15]. Cytokines and proteases, the architects of inflammation, not only activate immune cells but also concurrently induce ongoing bone resorption. Specific cytokines activate lymphocytes, driving increased osteoclast differentiation and activation, while impairing bone formation by osteoblasts. This intricate dance of immune signaling exacerbates bone loss, perpetuating the cycle of periodontitis [16,17]. Intriguingly, emerging evidence suggests that periodontal diseases might be influenced by gene expression. Scientists have employed cutting-edge techniques such as single-cell RNA sequencing (scRNA-seq) to delve deep into the immune microenvironment, unraveling diverse molecular mechanisms within the periodontium [18]. This advanced technology offers unprecedented insights into the intricate molecular dance underlying periodontal diseases.

In summary, periodontitis is a multifactorial disease initiated by the accumulation of dental plaque and influenced by a myriad of molecular mechanisms, immune responses, and potential genetic factors. A profound understanding of these intricate pathological processes is pivotal for formulating effective therapeutic and preventive strategies, safeguarding oral health, and enhancing the overall quality of life for affected individuals.

## 3. Pathogenesis of COVID-19: Exploring the Complex Interactions between SARS-CoV-2 and Host Receptors

Despite not being the first major pandemic of the 21st century, COVID-19 has garnered unprecedented global attention and response. COVID-19, caused by the SARS-CoV-2, is believed to have zoonotic origins and spreads rapidly among humans through respiratory droplets and contact [19,20]. SARS-CoV-2 possesses an enveloped positive single-stranded RNA structure, with a viral genome ranging from 8.4 to 12 kDa in size [19]. The genome contains a 5′ terminal region rich in open reading frames encoding proteins crucial for virus replication [19]. Conversely, the 3′ terminal region comprises five structural proteins: spike protein, membrane protein, nucleocapsid protein, envelope protein, and hemagglutinin-esterase protein [19]. In the intricate landscape of COVID-19 pathogenesis, angiotensin-converting enzyme 2 (ACE2) emerges as a pivotal player, serving as the primary receptor for SARS-CoV-2 [19,20]. Early in the infection process, ACE2 facilitates the attachment and entry of the virus into host cells. The interaction between the viral spike protein and ACE2 triggers critical conformational changes, leading to viral membrane fusion and the release of genetic material into the host cell. Initially recognized as a homologue of ACE, ACE2 has become a central focus in understanding SARS-CoV-2 infection dynamics [20]. Another key actor in this viral drama is transmembrane serine protease 2 (TMPRSS2), a membrane-bound serine protease that significantly influences COVID-19 pathogenesis [21]. In cell lines, SARS-CoV relies on endosomal cysteine proteases cathepsin B and L (CatB/L), as well as the TMPRSS2, for priming the S protein during viral entry. Blocking both proteases is necessary in effectively preventing viral entry. However, only TMPRSS2 activity is vital for viral spread and the progression of the infection within the host, while CatB/L activity is not essential for these processes [22]. The susceptibility of a host to SARS-CoV infection primarily hinges on the affinity between the viral receptor-binding domain (RBD) and host ACE2 during the initial attachment phase [23,24]. Organs rich in ACE2-expressing cells, such as respiratory epithelial cells, renal tissue, blood vessels, and cardiac tissues, emerge as high-risk targets for COVID-19 infection [25]. Moreover, the high expression of ACE2 in epithelial cells accentuates the vulnerability of these organs to viral invasion and COVID-19 pathology [26,27]. Recent research has spotlighted the potential involvement of the oral cavity and periodontal tissues in COVID-19 pathogenesis. ACE2 expression in the oral mucosa, tongue, and salivary glands suggests these tissues could serve as focal points for SARS-CoV-2 infection [28,29]. Additionally, the presence of ACE2 in gingival epithelium and periodontal ligament cells implicates periodontal pockets as potential contributors to viral transmission and disease progression [12].

In summary, the pathogenesis of COVID-19 unfolds as a multifaceted interplay between SARS-CoV-2 and host receptors, primarily ACE2 and TMPRSS2. ACE2 acts as the primary gateway for viral entry, while TMPRSS2 plays a crucial role in membrane fusion and viral infiltration into host cells [22,30]. The tissue-specific patterns of ACE2 expression confer susceptibility to specific organs, designating them as high-risk targets for viral invasion. Understanding these intricate interactions is fundamental for developing targeted therapeutic strategies against COVID-19.

## 4. Systemic Implications of Periodontitis: Bridging Oral Health to Overall Well-Being

Beyond its confines in the oral cavity, periodontal disease has emerged as a significant player in the broader spectrum of systemic health. Recent research endeavors have underscored the intricate interconnections between periodontal health and systemic diseases [31,32,33]. Within the gingival sulcus, a bustling microcosm of immune cells orchestrates a harmonious defense, constituting the body’s frontline protection mechanism. This immunological brigade not only guards the periodontium but also shapes our immune system’s initial response to external threats. The repercussions of periodontal disease reverberate through various systemic domains. Stroke, a devastating cerebrovascular event, was found to be correlated with periodontal disease in a previous study [34]. However, maintaining periodontal health through dental prophylaxis and periodontal disease treatment can help reduce the incidence of ischemic stroke [34]. This finding underscores the impact of oral health on overall well-being and emphasizes the importance of preventive measures in reducing the risk of serious conditions such as stroke. Respiratory infections, including pneumonia, are also unexpectedly connected, amplifying the significance of oral health in safeguarding respiratory well-being [35]. Premature mortality casts a shadow over those afflicted by periodontal disease, emphasizing the urgency of comprehensive oral care [36]. The entanglement between periodontitis and diabetes further highlights the systemic impact of oral health [32,33]. Studies have elucidated the bidirectional relationship between diabetes and periodontal disease, necessitating holistic management approaches for affected individuals [32,33]. The cardiovascular arena, too, bears the brunt of periodontal disease, with associations surfacing not only in myocardial infarction but also in conditions like abdominal aortic aneurysm, cardiovascular death, and atherosclerosis [37,38,39,40,41]. An increasing number of preclinical animal models and epidemiological studies undoubtedly indicate a close relationship between rheumatoid arthritis (RA) and periodontal disease [42]. Specifically, two crucial microorganisms, *P. gingivalis* and *A. actinomycetemcomitans*, play significant roles in the pathogenesis of both periodontal disease and RA, and their presence is associated with increased citrullination [42]. This heightened citrullination, the process of converting arginine to citrulline, is not only linked to RA but also participates in the tissue destruction observed in periodontitis. These findings underscore the intricate interconnection between these diseases, shedding light on potential avenues for comprehensive research and treatment strategies. Alzheimer’s disease (AD) is a global neurodegenerative challenge, affecting millions and becoming more prevalent as life expectancy rises [43]. Research suggests a mutual connection between Alzheimer’s and periodontitis [43]. Individuals with periodontitis have a higher likelihood of developing AD. Conversely, those with AD or dementia may struggle with oral care due to cognitive decline, increasing their vulnerability to chronic oral ailments like periodontitis [43]. This complex relationship underscores the significance of holistic healthcare addressing both neurological and oral health needs, particularly as the aging population grows. Pathogenic microorganisms, notably *P. gingivalis*, have been identified in both oral and systemic diseases, cementing its role as a significant causative agent in health issues spanning oral and systemic realms. Moreover, the link between persistent oral inflammation and oral cancer presents a concerning scenario, emphasizing the importance of thorough oral hygiene practices and routine screening efforts. In the realm of respiratory health, the intersection between lung diseases and periodontal health has been explored, unveiling connections that warrant serious consideration. Studies by Kaneoka et al. illuminated a troubling correlation between lung diseases, particularly pneumonia, and periodontal disease [44]. Pneumonia patients requiring extensive healthcare interventions not only face increased costs but also grapple with elevated mortality rates, underscoring the profound impact of oral health on overall health outcomes [44,45]. In the midst of this intricate interplay between periodontal health and systemic diseases, the emergence of COVID-19 further intensifies the spotlight on oral health. While COVID-19 primarily manifests as a respiratory illness, its symptoms echo the challenges faced in various respiratory conditions. Severe cases of COVID-19 demand intensive care, often involving critical oxygen support. The high case fatality rates among those hospitalized in intensive care units underscore the gravity of the situation, emphasizing the critical role oral health may play in overall susceptibility to severe outcomes [46].

As our understanding of these complex associations deepens, it becomes increasingly evident that oral health is not an isolated concern but an integral part of overall well-being. Both the prevention and management of periodontal disease should be approached with a sense of urgency, recognizing its profound impact on systemic health. Integrative healthcare strategies that bridge oral health to broader systemic health imperatives are imperative.

## 5. Recent Clinical Insights into the Intersection of Periodontitis and COVID-19

In the ever-evolving landscape of the COVID-19 pandemic, the relationship between periodontal diseases and the novel coronavirus has become a focal point of research. A noteworthy study conducted in India meticulously examined the short-term effects of COVID-19 on the tissues surrounding dental implants among local clinic patients. The results offered a sigh of relief, indicating no significant adverse effects on periodontal ligaments and gums in the short run. However, the shadows of uncertainty loom large concerning the long-term impacts, emphasizing the need for further, extensive research in this domain [47,48]. On the other hand, a systematic review and meta-analysis of 22 studies involving 92,535 patients from different regions found a significant association between periodontitis and adverse COVID-19 outcomes [49]. Patients with periodontitis are at higher risk for severe COVID-19 symptoms, ICU hospitalization, and death. Severe periodontitis is particularly associated with worse outcomes than mild periodontitis. However, due to methodological differences between studies, further standardized studies are needed to fully understand the relationship between periodontal health and COVID-19 outcomes [49]. The human microbiota encompasses a diverse range of microbial communities residing within the host, including bacteria, viruses, fungi, and protozoans. Surprisingly, the human body hosts more microbes than human cells, residing in mucosal membranes and on the skin. Extensive research has focused on understanding the vital role of the human microbiota in shaping immunity and maintaining homeostasis, with a particular emphasis on the gut, where these microbes are most abundant [50]. A preliminary study involving the sequencing of fecal samples from 15 patients indicated that COVID-19 infections have a substantial impact on fecal microbiomes. This impact is characterized by a decrease in beneficial commensal bacteria and an increase in opportunistic pathogens [51]. The study found that the initial levels of *Coprobacillus* and certain *Clostridium* species were linked to the severity of COVID-19. Additionally, the presence of *Faecalibacterium prausnitzii*, which possesses anti-inflammatory properties, was inversely related to the severity of COVID-19, highlighting the influence of SARS-CoV-2 on the gut microbiome [51]. However, while there is indirect evidence suggesting that probiotic supplementation might mitigate the severity of the COVID-19 response, including reducing mortality, numerous clinical trials are currently underway globally [50]. These trials aim to clarify the role of probiotics in both preventing and treating COVID-19. The pandemic did not merely cast a shadow on oral health but also influenced dental treatments significantly. The initial COVID-19 outbreak significantly reduced patient visits at private endodontic offices, leading to higher pain levels reported by patients. Older individuals were less likely to seek dental care, while those with kidney diseases were more inclined to visit. Cases with prior treatments decreased, and there was a notable increase in nonsurgical root canal treatments and apicoectomies, intensifying the burden on endodontic practices during the pandemic [52]. A study evaluated the impact of the COVID-19 pandemic on dental care at a university referral center in Madrid, Spain. A retrospective analysis of 392 medical records found that during the first wave of the epidemic, 58.67% of conservative treatments, 47.45% of periodontal treatments, 27.30% of clinical activities, and 13.52% of other treatments were not performed. Interestingly, age, gender, and nationality did not play a significant role in the absence of dental treatment. These findings highlight the potential negative impact of COVID-19 on basic treatments such as conservative care and periodontal surgery, potentially increasing the risk of tooth loss in adults [53]. In a groundbreaking study, researchers investigated the presence of SARS-CoV-2 in periodontal tissues of COVID-19 positive patients. Utilizing minimally invasive post mortem biopsies in seven fatal cases, the study identified SARS-CoV-2 RNA in periodontal tissues through RT-PCR analysis in five cases [54]. Histopathological examinations revealed morphological changes, confirming viral presence [54]. These findings provide crucial evidence of SARS-CoV-2 in periodontal tissues, highlighting potential oral transmission routes. Emerging strains of the SARS-CoV-2 are leading to increased infectiousness. Dental practitioners, especially oral medicine specialists, are constantly exposed to the virus due to direct contact with patients’ respiratory pathways, prolonged exposure during procedures, and certain treatments that generate aerosols. A new collaborative research project across Europe aims to evaluate the prevalence of oral lesions and associated risk factors in healthcare staff after COVID-19 vaccination [55]. The early identification of potential SARS-CoV-2 infections in individuals showing oral lesions without systemic symptoms could serve as a crucial safety measure for dentists, patients’ families, and colleagues.

In summary, the connection between COVID-19 and oral health is multifaceted. Short-term studies reveal immediate impacts on dental treatments, while long-term concerns necessitate ongoing research. Periodontitis is associated with severe COVID-19 outcomes, emphasizing oral health’s crucial role. Disruptions in dental care due to the pandemic have reshaped treatment approaches. The discovery of SARS-CoV-2 in periodontal tissues highlights potential oral transmission routes, underscoring the need for stringent preventive measures. Monitoring oral lesions post-vaccination is crucial. Overall, continuous research and collaborative efforts are essential for adapting dental practices and ensuring the safety of both patients and healthcare professionals amid the evolving challenges of the pandemic.

## 6. The Possible Mechanism Linking Periodontitis and COVID-19

The potential connection between periodontitis and COVID-19 has been the subject of numerous hypotheses [56]. In individuals affected by COVID-19, there is an observed increase in Th17 cells that produce interleukin-17 (IL-17) [56]. Remarkably, individuals suffering from periodontitis also display elevated levels of IL-17 [56]. This simultaneous elevation suggests a possible link between periodontitis and COVID-19. Notably, IL-17 has the ability to enhance osteoclast activity by stimulating the expression of RANKL on osteoblasts. Subsequently, RANKL binds to RANK on osteoclast precursors, promoting their transformation into mature osteoclasts. This process initiates osteoclast activity, leading to bone resorption [57]. COVID-19 is caused by the SARS-CoV-2, which requires ACE2 and TMPRSS2 for entry into host cells. ACE2 serves as the receptor for the virus, while TMPRSS2 acts as a protease [58]. The presence of ACE2 and TMPRSS2 in the oral cavity might amplify COVID-19 pathogenesis or worsen periodontitis. Apart from IL-17, ACE2, and TMPRSS2, a complex interrelation also exists involving COVID-19, periodontitis, and melatonin [59]. SARS-CoV-2 can be detected in the oral cavity and saliva, potentially heightening susceptibility to periodontitis and gingivitis resulting from COVID-19. The NLR family pyrin domain containing 3 (NLRP3) inflammasome plays a central role in the cytokine storm observed during both COVID-19 and periodontal inflammation [59]. The activation of the NLRP3 inflammasome can lead to the release of pro-inflammatory cytokines, causing tissue damage and inflammation in both conditions [59]. Significantly, melatonin has been found to inhibit the activation of the NLRP3 inflammasome, suggesting that melatonin treatment during COVID-19 or periodontal diseases could alleviate the damage observed in periodontal tissues [59]. Hence, melatonin emerges as a potential protective agent against both COVID-19 and periodontitis. Nevertheless, it is crucial to recognize that the precise mechanism linking periodontitis and COVID-19 remains elusive [60]. Consequently, further research is essential to comprehensively elucidate the pathogenesis of periodontitis and its intricate association with COVID-19. Ongoing studies in this field are necessary to uncover the intricate details of this relationship, shedding light on potential therapeutic interventions and preventive strategies for individuals susceptible to both conditions.

## 7. How to Lower the Risk of Susceptibility to Periodontitis and COVID-19

Reducing susceptibility to both periodontitis and COVID-19 involves a multifaceted approach, addressing shared risk factors and embracing proactive health practices. Aging stands as a common vulnerability, with elderly individuals facing heightened risks due to age-related health decline. In the context of COVID-19, older adults are more susceptible to severe outcomes, often necessitating intensive care interventions [61]. Likewise, in periodontitis, factors such as chronic diseases, inadequate oral hygiene, and a lack of dental care elevate vulnerability among the elderly [62]. Diabetes, a prevalent risk factor for both conditions, accentuates susceptibility. Individuals with diabetes face increased hospitalization rates and heightened severity when infected with COVID-19. Poor glycemic control escalates the risks, making effective diabetes management crucial in mitigating these complications [63]. Similarly, diabetes significantly amplifies the risk of periodontitis, underscoring the importance of comprehensive oral health management for diabetic individuals [64]. Chronic inflammation, a common thread intertwining both diseases, complicates recovery in post-COVID cases and can contribute to conditions like long COVID. This low-grade inflammation (LGI) also mirrors the chronic inflammatory environment seen in periodontitis and highlights the intricate interplay between systemic inflammation and oral health [65,66]. To mitigate the risk of periodontitis, adopting meticulous oral hygiene practices is paramount. Regular tooth and tongue brushing, complemented by flossing and dental visits, form the cornerstone of preventive oral care [67]. The choice of a soft-bristled toothbrush, whether manual or electric, is pivotal in plaque removal while minimizing mucosal trauma. Fluoride toothpaste plays a pivotal role in maintaining oral hygiene standards. Additionally, interdental cleaning aids like dental floss, interdental brushes, or dental sticks should be utilized, guided by healthcare professionals to ensure proper technique [68]. Importantly, research underscores the significance of mechanical oral cleaning in preventing healthcare-associated pneumonia, emphasizing the integral role of oral care in broader health contexts [44]. Hence, promoting effective oral hygiene practices not only preserves dental health but also contributes significantly to overall health outcomes, especially for individuals susceptible to both periodontitis and COVID-19.

## 8. Discussion

The nexus between periodontitis and COVID-19, as explored in this comprehensive review, brings forth a multifaceted perspective, intertwining the intricate complexities of oral health with the challenges posed by a global viral pandemic. While direct causative links between periodontitis and severe COVID-19 outcomes are yet to be unequivocally established, the confluence of biological factors and shared pathways unearthed in this exploration underscores the need for a deeper understanding of these interactions.

One of the pivotal observations in this study is the presence of ACE2 receptors in oral epithelial tissues [27]. This revelation amplifies the significance of oral health in the context of COVID-19 susceptibility. Periodontal pockets, often rife with inflammation and compromised tissue integrity, might serve as vulnerable entry points for the SARS-CoV-2 [12]. This highlights the urgent need for meticulous oral hygiene practices, not only for the preservation of dental health but also as a potential means of reducing viral exposure. Furthermore, the impact of periodontal disease on systemic health, including conditions like diabetes and heart disease [37,39], underscores the broader implications of oral health management in mitigating the severity of COVID-19. A comprehensive approach to healthcare must encompass oral health assessments, especially for individuals predisposed to systemic conditions, ensuring a holistic understanding of their vulnerability to severe viral infections.

The convergence of IL-17, a cytokine associated with bone loss, in both periodontitis and COVID-19 signifies a shared immunological pathway [56]. This commonality hints at the potential for interventions targeting this pathway to have dual benefits. Strategies aimed at modulating IL-17 levels could not only aid in managing periodontal disease but also potentially mitigate the impact of COVID-19 on bone health. This intersection of therapeutic opportunities underscores the importance of interdisciplinary research, bringing together specialists from diverse fields to explore innovative solutions that transcend traditional medical boundaries. Additionally, the involvement of the NLRP3 inflammasome in both periodontitis and COVID-19 paints a complex immunological landscape [59]. The ability of melatonin to inhibit the activation of the NLRP3 inflammasome adds a promising therapeutic dimension [59]. Melatonin, a hormone with well-documented roles in regulating sleep patterns, emerges as a potential guardian against the damaging effects of inflammation in both oral tissues and vital organs affected by COVID-19 [59,69]. Further studies investigating the efficacy of melatonin-based interventions could pave the way for novel therapeutic approaches, not only in managing periodontitis and COVID-19 but also in addressing other inflammatory conditions.

Yet, amidst the promising avenues illuminated by this review, critical gaps in knowledge persist. Long-term studies tracking the oral health trajectories of COVID-19 survivors are essential. These investigations could offer insights into the potential lingering effects of the virus on periodontal tissues, shedding light on post-recovery complications that might impact oral health. Additionally, a nuanced understanding of the specific mechanisms through which periodontal tissues facilitate viral entry remains crucial. Detailed molecular studies dissecting the interactions between viral particles and oral epithelial cells could unravel the intricacies of this process, providing a foundation for targeted interventions. In the realm of public health, the integration of oral health into broader healthcare frameworks becomes imperative. Public awareness campaigns focusing on the significance of oral hygiene in the context of viral susceptibility are essential. Individuals, especially those with pre-existing systemic conditions, need to be educated about the potential implications of periodontitis on COVID-19 outcomes. Healthcare professionals, ranging from dentists to infectious disease specialists, must collaborate to ensure comprehensive patient care. Regular oral health assessments, especially for vulnerable populations, should be a standard practice, empowering individuals to safeguard their oral health as a vital component of their overall well-being.

## 9. Conclusions

In conclusion, this review emphasizes the dynamic interplay between periodontitis and COVID-19, illuminating promising avenues for future research and clinical interventions. As the scientific community delves deeper into the intricacies of these interactions, collaborative efforts between oral health specialists, immunologists, and infectious disease researchers become paramount. This interdisciplinary synergy not only expands our understanding of the complex relationship between oral health and viral infections but also offers a foundation for innovative and targeted interventions. By addressing the challenges posed by periodontitis and COVID-19 in a unified manner, healthcare systems can fortify individuals against the multifaceted impacts of these conditions, ensuring a resilient and healthier global population.

## Data Availability

The data presented in this study are available in this article.

## Abbreviations

COVID-19	Coronavirus disease 2019
SARS-CoV-2	Severe acute respiratory syndrome coronavirus 2
GCF	Gingival crevicular fluid
CTSS	Cathepsin S
scRNA-seq	Single-cell RNA sequencing
ACE2	Angiotensin-converting enzyme 2
TMPRSS2	Transmembrane serine protease 2
RBD	Receptor-binding domain
AD	Alzheimer’s disease
IL-17	Interleukin-17
NLRP3	NLR family pyrin domain containing 3
LGI	Low-grade inflammation
RA	Rheumatoid arthritis
CatB/L	Cathepsin B and L
